# Painful Ejaculation - An Ignored Symptom

**DOI:** 10.7759/cureus.11253

**Published:** 2020-10-30

**Authors:** Muhammad Waqar, Kawa Omar, Amr Moubasher, Oliver Brunckhorst, Kamran Ahmed

**Affiliations:** 1 Urology, King's College Hospital NHS Foundation Trust, London, GBR; 2 Urology, MRC Centre for Transplantation, Guy's Hospital Campus, King's College London, King's Health Partners, London, GBR

**Keywords:** post-orgasmic pain, painful ejaculation, dysejaculation, odynorgasmia, post orgasmic pain, dysorgasmia

## Abstract

The purpose of this review is to summarize the pathophysiology of ejaculation and look into prevalence, aetiology, diagnosis, and treatment of painful ejaculation. We carried out a comprehensive search of PubMed in order to look for literature on male painful ejaculation using keywords post-orgasmic pain, painful ejaculation, dysejaculation, odynorgasmia, post-orgasmic pain, or dysorgasmia.

Painful ejaculation has an alarming prevalence throughout the world, between 1 to 25%. It has a detrimental effect on patients’ quality of life as it reduces individual self-esteem and is associated with sexual dysfunction. Its aetiology includes simple infection or inflammation of the urinary tract, benign prostate hyperplasia, ejaculatory duct obstruction, post-radical prostatectomy and side effects of certain medications. Once reported, it should be investigations and treatments should be tailored according to the etiology. Both medical and surgical treatment is available depending on the cause of painful ejaculation.

Due to the sensitive nature of its presentation, it is a symptom that can be identified best when specifically asked. Our understanding regarding painful ejaculation is very limited and only a few articles have revealed insight into this topic. Further research is required in order to set proper guidelines for diagnosis and treatment of painful ejaculation.

## Introduction and background

Orgasm-associated pain is defined as an agonising sensation occurring during orgasm. It has been mainly reported at the level of the penis, but pain at various sites including testes, rectum, or the lower abdomen had been reported [1]. It is also known as dysejaculation, odynorgasmia, dysorgasmia, or orgasmalgia [2]. This is a poorly understood phenomenon, therefore mostly ignored. The incidence of orgasm-associated pain has been reported from 1.9-25% [3-4]. The duration of the pain can be a few seconds or persist up to two days [5-6]. The sensation can vary from a dull ache to unbearable pain [7]. Unfortunately, relatively few articles have shed light on this topic. The objective of this literature review is to find the incidence, causes, investigations, and potential management for painful ejaculation.

## Review

Prevalence

Painful ejaculation remains an under-reported symptom. A large-scale, multinational survey was carried out in the United States and six European countries in 2003, which looked into 12,815 male patients ranging from 50 to 80 years of age suffering from lower urinary tract symptoms (LUTS). Of these patients, 6.7% were suffering from painful ejaculation [8]. In another study, 25.9% of approximately 2000 sexually active men with LUTS, had been experiencing ejaculatory discomfort. In another two studies, the incidence was reported to be from 1.9 to 12% [3,9]. Thus, the prevalence ranges from 1.9 to 25% of men either as an isolated symptom or associated with other lower urinary tract symptoms. Moreover, a rise in the number of cases was noted with increased severity of LUTS assessed by international Prostate Symptom Score [4].

Orgasm physiology

**Figure 1:**
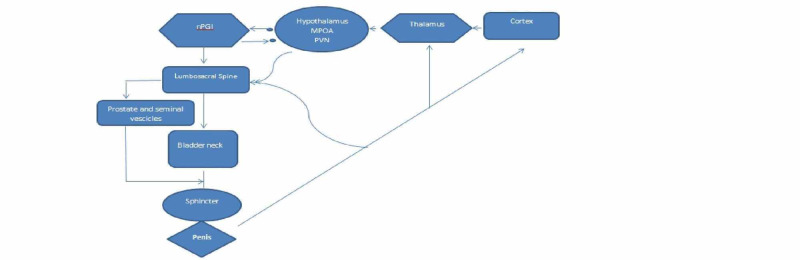
Physiology of orgasm and ejaculation. MPOA: medical preoptic area, PVN: paraventrucular thalamic nucleus, nPGI: paragigantocellularis nucleus.

Understanding the physiology of orgasm is crucial in comprehending painful ejaculation. In men, orgasm and ejaculation happen simultaneously. Ejaculation has two stages, emission and ejection [10]. Ejaculation and orgasm depend on a perplexing interplay between the central nervous system and the peripheral nervous system, with the inclusion of some neurotransmitters, namely dopamine, norepinephrine, serotonin, acetylcholine, and nitric oxide. Hormonal pathways also play a significant role in ejaculation. Typical hormones involved are oxytocin, prolactin, thyroid hormone, glucocorticoids, and sex hormones. Unfortunately, very few studies have evaluated hormones effect on ejaculation physiology [11-12]. Brain studies assessing ejaculation and orgasm have shown a significant role of the thalamus and hypothalamus in controlling sexual behavior [13,11]. In Figure 1 this connection has been demonstrated [1]. The sensory input from the dorsal nerve of the penis transmits sensation to the spinal cord. Stimulation of perineum and testes also activate the dorsal nerve resulting in similar effects [14].

Emission and expulsion are the two phases of ejaculation. The initial step of the ejaculation procedure is the outflow stage, also called the emission phase. This starts by closure of the bladder neck preventing backflow of secretions. The epididymis, vas deferens, original vesicles, prostate organ, prostatic urethra, and bladder neck are all involved in this stage. This is followed by the ejection of prostatic secretion and spermatozoa from the vas deferens into the prostatic urethra. During the emission phase, both the pelvic and the hypogastric plexus play a vital role after receiving physical or visual stimulation [15]. This is continued by the ejection of semen through the urethral meatus. This is called the expulsion phase and is characterized by contractions of different striated muscle including bulbospongiosus and ischiocavernosus muscles [16]. Throughout the process, the bladder neck remains closed. The relaxation of the external urinary sphincter and activation of pudendal nerve fiber along with other accessory sexual organs are processed at the level of the brain, thus inducing the sensation of orgasm [12].

Aetiology

There are a multitude of factors implicated in painful ejaculation. Though few are life-threatening, painful ejaculation can significantly impact the individual’s quality of life. Below we have listed the common possible causes.

Infectious or inflammation: Conditions such as orchitis, epididymitis, prostatitis, or urethritis have been found to cause painful ejaculation [17-18].

Benign prostate hyperplasia: Litwin study showed patients with benign prostate hyperplasia (BPH) were more prone to have painful ejaculation than the normal population [19].

Post radical prostatectomy: Intra-operative damage to the bladder neck and nerve fibre controlling bladder neck contraction and external sphincter muscle leads to orgasm related symptoms. In one study, it was shown that 9% of patients treated with radical prostatectomy suffered from painful ejaculation [12]. In another study, 33% experienced painful ejaculation post-radical prostatectomy [20].

Seminal vesicle stone: The cause of seminal vesicle stones is still a mystery, but it is seen in patients with chronic infection, prostate cancer, reflux of urine, or diabetes mellitus. The majority of these patients present with painful ejaculation and hematospermia. Diagnosis is made by radiological assessment such as trans-rectal ultrasound and magnetic resonance imaging, MRI, or plain radiographs [21].

Zinner syndrome: It is a triad of ipsilateral renal agenesis, seminal vesicle cysts and ejaculatory duct obstruction. Seminal vesicle cysts are found in 70% of cases of ipsilateral renal agenesis. It is mostly an incidental finding but sometimes patients can present with painful ejaculation [22].

Ejaculatory duct obstruction (EDO): It is a rare condition that can be caused by several pathologies such as ejaculatory duct malformations, midline prostatic cysts, fibroses due to prostatitis or seminal vesiculitis, seminal vesicle (SV) stones, or scarring after endoscopic manipulation. EDO may present with painful ejaculation co-existing with infertility. This group of patients is difficult to manage due to their complex anatomy [23-25].

Chronic pelvic pain syndrome: Pudendal neuropathy has been mostly caused by nerve compression and can result in painful ejaculation [6]. Other areas of pain include the penile, scrotal, and peri-anal region. It is thought to be due to pelvic movements during intercourse. The damage mostly happens as the pudendal nerve passes between the sacrotuberous and sacrospinous ligaments [26]. Twenty-four percent of patients suffering from chronic pelvic pain syndrome (CPPS) were found to have ejaculatory pain regularly [27].

Medication: Painful ejaculation is also seen with anti-depressant drugs. It was noticed with imipramine, desipramine, clomipramine, protriptyline, amoxapine, fluoxetine, and venlafaxine [28]. Muscle relaxants such as cyclobenzaprine was also reported to cause painful ejaculation [29]. Stopping these medications have shown improvement in patient symptoms.

Miscellaneous: Ejaculatory pain after vasectomy is rare but if it happens, it usually occurs at the scrotum [30]. Scarring of the vas deferens following inguinal hernia repair with mesh can also result in painful ejaculation [31]. It mainly results from migration of mesh resulting in permanent damage to nerve, vas deference, and spermatic cord.

**Figure 2:**
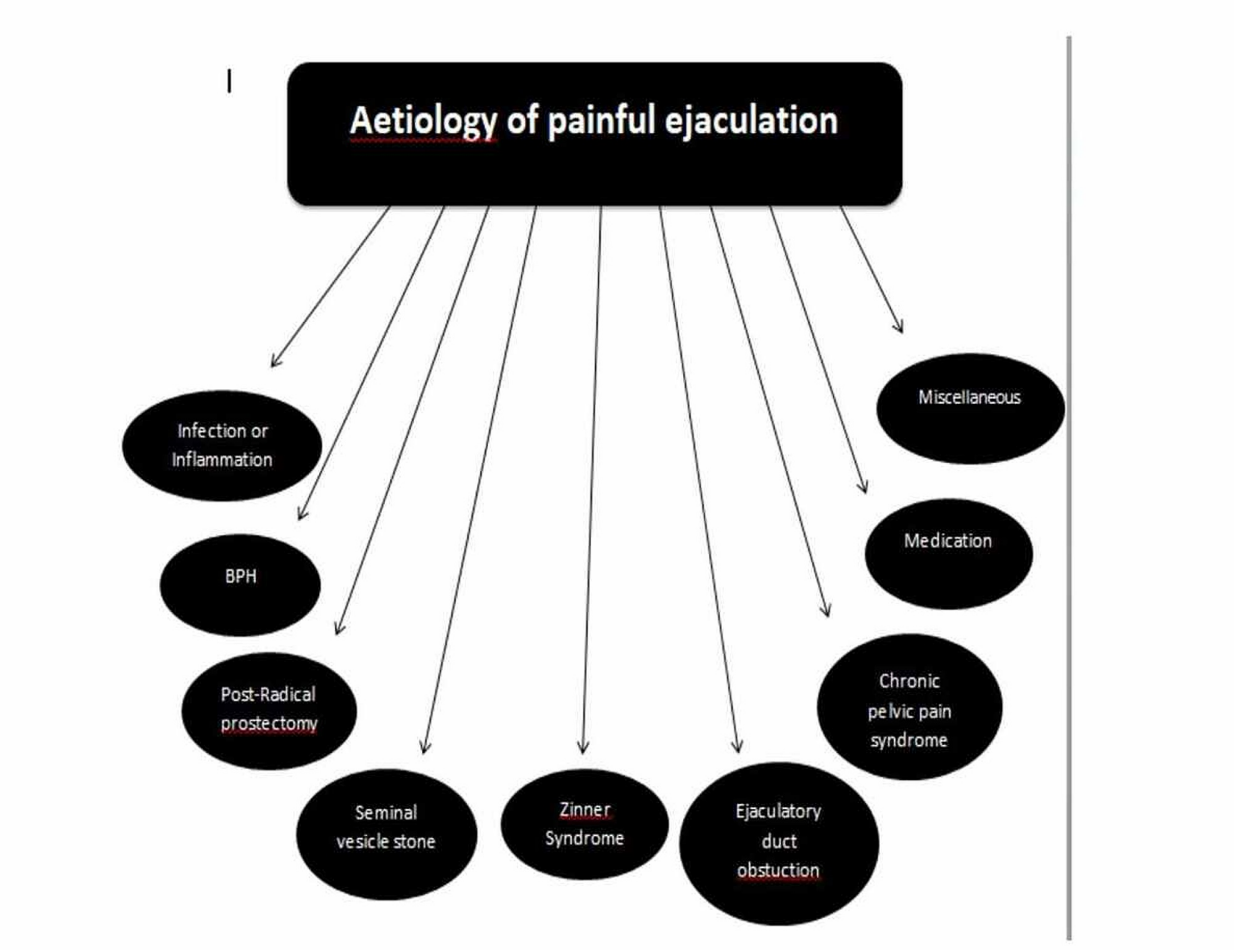
Aetiology of painful ejaculation BPH: benign prostate hyperplasia

Figure 2 summarizes the causes of painful ejaculation.

Investigations required are tailored to the suspecting cause of the painful ejaculation. Assessment of these patients includes a full history and physical examination including genitals and digital rectal examination for prostate. Investigation ranges from urinalysis, urine culture, and blood tests including PSA to trans-rectal ultrasound to check for ejaculatory duct obstruction or calculi. Cystoscopy can be performed if urethral stricture was suspected or urethrogram can be offered instead [3].

Treatment

Treatment depends upon the cause of post-orgasmic pain. If the cause is suspected to be infectious or inflammatory processes, antibiotics and non-steroidal anti-inflammatory drugs are used. For seminal vesicle related pain, transurethral seminal vesiculoscopy is the approach of choice [21]. In ejaculatory duct obstruction, transurethral resection of the ejaculatory duct or balloon dilatation can resolve the issue [32]. In one study, patients treated with tamsulosin for four weeks showed a significant improvement in symptoms [5]. This is also useful in post-radical prostatectomy related painful ejaculation [33]. In an article by Perez et al. [34], a young patient with post ejaculation pain was successfully treated with oral topiramate. Within a month, the patient’s pain score improved from 8/10 to 1/10. Conventional analgesics and neuropathic pain therapies failed to eliminate ejaculatory pain. Painful ejaculation due to medication side effects can be controlled by stopping the medication. In the case of post inguinal hernia ejaculatory pain, exploration of wound and releasing the vas deferens from the scar tissue and dividing the ilioinguinal nerve proved to alleviate the pain [7]. Table 1 summarizes the treatment of painful ejaculation as per the aetiology.

**Table 1:** Treatment as per aeitology. BPH: benign prostate hyperplasia

Condition/Aeitology	Treatment
Infection (orchitis, epididymitis, prostatitis, or urethritis)	Antibiotic
BPH	Tamsulosin
Post radical prostatectomy	Tamsulosin
Seminal vesicle stone	Transurethral seminal vesiculoscopy and removal of stone
Anti-depressant drugs	Stop medication
Post inguinal hernia ejaculation pain	Release vas deferens from scar and divide ilioinguinal nerve
Ejaculation duct obstruction	Transurethral resection of ejaculatory duct or balloon dilation

## Conclusions

Painful ejaculation is an underreported symptom that has a detrimental effect on patients’ quality of life and therefore should be actively identified by clinicians. It remains an often ignored symptom. It’s a symptom that requires specific interrogation, with many patients often not volunteering their experiences at first. It has multiple causes and clinicians should specifically ask for it. Once reported, investigations and treatments should be tailored according to the etiology. Treatment varies from withdrawal of suspected agent (drugs) or prescribing right medical treatment (antibiotic, anti-inflammatory, or antibiotic). Surgical option is also available depending on etiology in form of resection of prostate, ejaculatory duct and neuolysis to correct pudendal neuropathy. Currently there are no specific guidelines on painful ejaculation by major urology bodies such as the American Urological Association (AUA), European Association of Urology (EAU), and NICE guidelines. This area that needs more research in order to have clear guidelines regarding its diagnosis and management.
